# Inferred relatedness and heritability in malaria parasites

**DOI:** 10.1098/rspb.2010.0196

**Published:** 2010-04-14

**Authors:** Tim J. C. Anderson, Jeff T. Williams, Shalini Nair, Daniel Sudimack, Marion Barends, Anchalee Jaidee, Ric N. Price, François Nosten

**Affiliations:** 1 Southwest Foundation for Biomedical Research (SFBR), San Antonio, TX, USA; 2 Shoklo Malaria Research Unit (SMRU), Mae Sot, Tak, Thailand; 3 Menzies School of Health Research, Charles Darwin University, Darwin, Australia; 4 Faculty of Tropical Medicine, Mahidol University, Bangkok, Thailand; 5 Centre for Tropical Medicine and Vaccinology, Churchill Hospital, Oxford, UK

**Keywords:** artemisinin, heritability, drug resistance, clearance rate, twins, clones

## Abstract

Malaria parasites vary in phenotypic traits of biomedical or biological interest such as growth rate, virulence, sex ratio and drug resistance, and there is considerable interest in identifying the genes that underlie this variation. An important first step is to determine trait heritability (*H*^2^). We evaluate two approaches to measuring *H*^2^ in natural parasite populations using relatedness inferred from genetic marker data. We collected single-clone *Plasmodium falciparum* infections from 185 patients from the Thailand–Burma border, monitored parasite clearance following treatment with artemisinin combination therapy (ACT), measured resistance to six antimalarial drugs and genotyped parasites using 335 microsatellites. We found strong relatedness structure. There were 27 groups of two to eight clonally identical (CI) parasites, and 74 per cent of parasites showed significant relatedness to one or more other parasites. Initially, we used matrices of allele sharing and variance components (VC) methods to estimate *H*^2^. Inhibitory concentrations (IC_50_) for six drugs showed significant *H*^2^ (0.24 to 0.79, *p* = 0.06 to 2.85 × 10^−9^), demonstrating that this study design has adequate power. However, a phenotype of current interest—parasite clearance following ACT—showed no detectable heritability (*H*^*2*^ *=* 0–0.09, ns) in this population. The existence of CI parasites allows the use of a simple ANOVA approach for quantifying *H*^2^, analogous to that used in human twin studies. This gave similar results to the VC method and requires considerably less genotyping information. We conclude (i) that *H*^2^ can be effectively measured in malaria parasite populations using minimal genotype data, allowing rational design of genome-wide association studies; and (ii) while drug response (IC_50_) shows significant *H*^2^, parasite clearance following ACT was not heritable in the population studied.

## 1. Introduction

Measurement of heritability (*H*^2^)—the degree to which a phenotypic trait is determined by genotype—is central to quantitative genetic analysis (Falconer & Mackay 1996). For example, traits with high *H*^2^ are expected to respond rapidly to selection, and mapping of genes that underlie traits with high *H*^2^ requires smaller sample sizes than for traits with weak heritability. Accurate measures of trait *H*^2^ can be made in organisms that are readily crossed in the laboratory and reared in common environmental conditions (Falconer & Mackay 1996). Alternatively, heritability can be measured using pedigrees (Kruuk *et al*. 2000; Havill *et al*. 2010). However, for many organisms, genetic crosses are difficult to perform and reliable pedigree information is not available. Furthermore, for many biomedical traits of interest, measurement in a controlled laboratory situation is impossible. For example, biologists working on malaria are interested to know why some parasite genotypes cause disease in humans, while others do not (Doumbo *et al.* 2009). In this case, disease severity is only possible to observe in humans and cannot be evaluated experimentally in the progeny of genetic cross. For this and other phenotypes, heritability should ideally be measured in natural populations.

One possible approach to measuring *H*^2^ in natural populations involves inferring relatedness using genetic markers, as individuals sharing alleles at multiple loci are more likely to be closely related than individuals sharing alleles at few loci (Thompson 1974; Ritland 2000; Blouin 2003; Csillery *et al*. 2006). There are two general approaches to doing this. First, identity-by-state (IBS) allelic information at each locus can be used to infer groups of related individuals (i.e. sibs, half-sibs, etc.), and then patterns of phenotypic variation within and between these groups can be used to determine *H*^2^ (Thomas *et al*. 2000; Fernandez & Toro 2006). Second, allele-sharing measures can be used to estimate relatedness between pairs of individuals, without categorizing pairs into particular relationship classes (Ritland 1996, 2000; Klaper *et al*. 2001). However, all available methods are constrained by levels of relatedness estimable within natural populations, which is limited for many organisms (Csillery *et al*. 2006; Shikano 2008).

Malaria parasites are haploid protozoans with mixed mating systems. Inbreeding predominates in low transmission regions (Paul *et al*. 1995; Anderson *et al*. 2000) and related or clonally identical (CI) parasites are frequently sampled within populations. Malaria parasites show extensive variation in many traits of biomedical or biological interest, including drug resistance (Fidock *et al*. 2008), growth rate (Reilly *et al*. 2007), virulence (Mackinnon *et al*. 2002) and sex ratio (Read *et al*. 1992; West *et al*. 2001). Loci underlying some malaria traits have been mapped effectively using linkage analysis, but genetic crosses required for such studies are cumbersome, expensive and unsuitable for many clinically related traits (Su *et al*. 2007). Furthermore, detailed SNP maps, and the potential for whole-genome sequencing of populations of parasites, have led to excitement about mapping the genes underlying these traits by genome-wide association (Su *et al*. 2007). For efficient design of such studies, it would be ideal first to know whether the traits of interest have a genetic basis and to use the extent of *H*^2^ to estimate appropriate sample sizes.

We evaluate the use of inferred relatedness for estimating *H*^2^ in natural malaria parasite sampled from a single clinic on the Thailand–Burma border. We use two different approaches. First, we inferred relatedness and phenotypic similarity between all pairs of parasites and generated estimates of *H*^2^ using variance components (VC) methods (Blangero *et al*. 2001). Second, we used only identical multilocus genotypes recovered from different patients, and estimated *H*^2^ using ANOVA, with methods analogous to those used in studies of human twins or clonal plants (Lynch & Walsh 1998). The VC approach models the contribution of *additive* variation only and ignores dominance effects or epistasis, so we are technically measuring *narrow-sense* heritability (*h*^2^) with this method. In contrast, the ANOVA method estimates the *broad-sense* heritability (*H*^2^), because genetic variation among CI parasites can be both additive and epistatic. We have used the notation for broad-sense heritability (*H*^2^) throughout the manuscript for both methods, both for clarity and because dominance is not relevant to haploid organisms such as blood-stage malaria.

We examined two different types of traits. Drug resistance is known to have a strong genetic basis (Hayton & Su 2004) and so provides an important positive control for our methods. We also investigated a trait of current topical interest: parasite clearance rate (CR) following treatment with artemisinin-based combination therapies (ACTs; Dondorp *et al*. 2009). ACTs are the first-line treatment for malaria in the majority of malaria endemic countries and result in extremely rapid clearance of parasites from the blood. Typically, 95 per cent of patients are parasite-negative by day 2 of treatment (White 2008). However, considerably slower clearance has been observed in some southeast Asian locations, leading to concern about the development of resistance (White 2008; Dondorp *et al*. 2009). It is currently unclear to what extent variation in CR is determined by parasite genetics or to patient or environmental factors. Estimates of *H*^2^ for this trait are of particular interest as genome-wide association studies (GWASs) have been proposed to try to identify the parasite genes that underlie variation in CR. Evidence of strong heritability for this trait comes from a recent study of Cambodian parasites (Anderson *et al*. 2010).

## 2. Material and methods

### (a) Collection of parasites

We collected 5 ml of *Plasmodium falciparum*-infected blood samples with greater than 0.5 per cent parasitaemia from patients visiting the malaria clinic at Mawker-Thai on the Thailand–Burma border, prior to treatment with ACTs. This clinic serves people on both sides of the border; 90 per cent of patients travel from within a 10 km radius around the clinic (F. Nosten 2004, personal communication). We excluded patients who (i) had taken malaria treatment within 60 days, (ii) were pregnant, or (iii) were co-infected with *P. vivax*.

### (b) Measurement of parasite clearance

Thin smears were collected prior to parasite treatment and at 24 and 48 h post-treatment for all patients. We measured the parasite reduction ratio (PRR), defined as (1 + parasite density_24 or 48 h_)/(1 + parasite density_admission_). For the subset of patients monitored every 6 h, we measured the first-order CR. This was quantified by plotting the natural log of parasite density against time since treatment in hours and measuring the slope.

### (c) Measurement of *in vitro* resistance

Infected blood samples were transported to the laboratory in Mae Sot within 4 h of collection, where we measured *in vitro* response to six drugs using a 48 h [^3^H] hypoxanthine incorporation assay (Desjardins *et al*. 1979; Brockman *et al*. 2000). This test measures growth by assaying incorporation of [^3^H] hypoxanthine in parasites cultured in 96-well plates. The drugs tested were chloroquine (CQ), quinine (QN), mefloquine (MFQ), lumefantrine (LUM), artesunate (AS) and dihydro-artemisinin (DHA). Tests were performed in duplicate at each of 11 doubling drug concentrations using a starting parasitaemia of 0.5 to 1 per cent (Anderson *et al*. 2005).

### (d) Microsatellite genotyping

We initially genotyped seven microsatellite loci to identify and exclude infections containing multiple clones (Anderson *et al*. 2005). We determined relationships between infections containing a predominant single clone by genotyping microsatellite markers spaced at approximately 50 kb intervals across the genome. We genotyped 15 parasites twice to measure genotyping reproducibility: only markers that showed 100 per cent reproducibility in comparisons of the 15 duplicated samples were included in the dataset. Marker positions, oligos and genotyping methods are listed in electronic supplementary material, table S1.

### (e) Measurement of relatedness

Relatedness is generally estimated for diploid species (Blouin *et al*. 1996). This study differs in that we are interested in relatedness between blood-stage parasites, which are the haploid products of meiosis. This is akin to measuring relatedness between gametes. We measured the proportion of shared alleles (ps) between all pairwise combinations of parasites and plotted 1−ps as a UPGMA tree using PHYLIP (Felsenstein 1993). Parasites that differed at less than 5 per cent of loci were assumed to be identical-by-descent (IBD) and are referred to as being clonally identical (CI). We compared frequency distributions of observed pairwise allele sharing with expectations from randomly generating unrelated parasites using the observed allele frequencies. In addition, we simulated expected allele sharing for haploid parasites derived from the same zygote, and for parasites derived from zygotes with one common parent. These simulations were performed using the observed allele frequencies at each locus using PopTools v. 3.2.0. (http://www.cse.csiro.au/poptools/index.htm).

### (f) Heritability *(H*^2^*)* estimation

We measured *H*^2^ using two different methods.

#### (i) VC *H*^2^ estimate

The VC approach uses relatedness between *all* pairs of parasites in the dataset to estimate *H*^2^ and is equivalent to the methods used for the analysis of multi-generational family studies of humans (Blangero *et al*. 2001). We used the proportion of shared alleles (Blouin 2003) as a simple metric of relatedness. Pairs of parasites that are genetically identical will share alleles at all loci, while pairs of parasites that are unrelated will share just a few alleles that are IBS by chance. Conventional VC methods for pedigree-based linkage analysis were used, except that matrices of inferred relatedness were used instead of the kinship matrix derived from pedigree data. For *H*^2^ estimation, the parasite phenotypic covariance matrix was modelled as 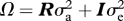, where ***R*** is the relationship matrix (equal to twice the kinship matrix), ***I*** is an identity matrix, 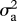 is the variance contributed by additive genetic factors and 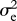 is the remaining (environmental and unmodelled genetic) variance. *H*^2^ is then estimated as 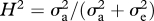. We used this procedure to estimate *H*^2^ of both log-transformed measures of *in vitro* drug resistance, PRR_24_ and PRR_48_.

#### (ii) ANOVA-based estimate

For this analysis, we used only CI parasite genotypes recovered from two or more individuals. We compared the variance of CR and IC_50_ phenotypes within and among clonal lineages of parasites, and estimated *H*^2^ from the mean-squares terms in the ANOVA as described in Lynch & Walsh (1998). In brief, we determined the within- and among-clone mean squares (*M*_Se_ and *M*_Sb_) for each trait. The total genetic variance 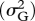 is estimated as 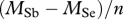. *n* is the weighted mean number of patients infected with each CI genotype and is calculated as follows: 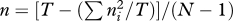, where *T* is the total number of patients, *N* is the number of different clones and *n*_*i*_ is the number of patients infected with the *i*th clone. The environmental variance 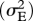 is estimated from the within-clone variation as *M*_Se_, and so 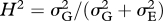.

### (g) Evaluating the effects of treatment type, patient age and patient gender

Patient age, patient gender and treatment type may potentially influence phenotype and were therefore included in the analysis. Each patient was treated with one of five different ACT treatment regimens. These five ACTs were divided into three groups for analysis: (i) group 1: mefloquine hydrochloride (8 mg kg^−1^) + artesunate (4 mg kg^−1^) for 3 days (MAS3) or 7 days (MAS7); (ii) group 2: dihydroartemisinin 7 mg kg^−1^/piperaquine 56 mg kg^−1^ body weight into four doses at 0, 8, 24 and 48 h (DP4) or into three daily doses (DP3); and (iii) group 3: fixed dose combination tablets (20 mg of artemether/120 mg of lumefantrine; Coartem, Novartis, Basel, Switzerland) as six doses over 3 days, administered by patient weight (COA6a). We evaluated the influence of these three independent variables on PRR_24_, PRR_48_, CR and IC_50_. To correct for the effects of these covariates, we used residuals from the regression analysis to examine *H*^2^.

## 3. Results

### (a) Relationships between parasite isolates

**Figure 1.**
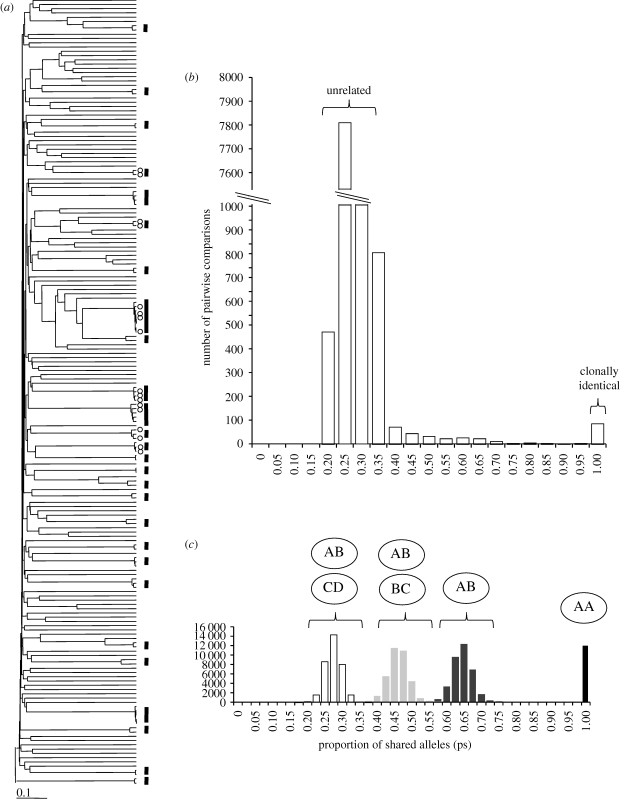
Relatedness structure of the parasite population. (*a*) UPGMA tree showing the relationships between 185 parasite isolates. The tree is constructed from a pairwise matrix of the statistic (1*–*ps), where ps is the proportion of alleles shared between the two isolates. These measures were calculated using 335 microsatellite markers genotyped. The bars mark 27 groups of parasites that differ by less than 5 per cent and are assumed to be IBD. The dots indicate the members of seven groups of parasites that differ by less than 5 per cent among the 61 parasites with detailed clearance data. (*b*) Pairwise measurements of allele sharing (ps). The *y*-axis is truncated to effectively display the range of relatedness within the population. (*c*) Simulated distribution of expected allele sharing for parasites in different relatedness classes. We simulated allele sharing expected in parasites derived from the same inbred oocyst (AA), from the same outcrossed oocyst (both from AB), from two related (half-sib) oocysts (from AB and BC) and from two unrelated oocysts (AB and CD). The observed ps distribution demonstrates that parasites are predominantly unrelated with some CI parasites and contributions from other relatedness classes.

The analysis is based on 185 infections containing a single parasite clone, for which measures of clearance, IC_50_ for six drugs and microsatellite genotype data (335 markers) are available. The microsatellite loci were highly polymorphic with 1–23 (mean = 11.04, median = 11, s.d. = 3.95) alleles per locus and expected heterozygosity of 0–0.92 (mean = 0.74, median = 0.79, s.d.=0.16). No more than seven (3.7%) genotypes were missing at any locus. The relationships between the parasites are summarized in figure 1. We found 27 clusters comprising two to eight parasites that differ by less than 5 per cent and are effectively IBD across the genome. These are referred to as CI genotypes. The few differences observed are distributed across the genome rather than in blocks. They are therefore most likely to be due to genotyping error and/or mutation. For the 61 (33%) patients for whom detailed 6-hourly clearance data were available, there were seven clusters of CI parasites, with two to four parasites per cluster (figure 1*a*).

Figure 1*b* shows the distribution of allele sharing between all pairwise comparisons for the 185 parasites (30 340 comparisons). The main peak is situated around 0.20–0.35. There is a tail to the right of this, suggesting parasites of intermediate relatedness. Finally, the peak at 0.95–1.0 shows parasite pairs that are CI. For comparison, we plotted the simulated distributions of allele sharing for different relatedness classes (figure 1*c*). The main peak in the observed data corresponds precisely with allele sharing expected for unrelated genotypes. We used the upper value of the simulated distribution for unrelated parasites (0.3556) to evaluate the proportion of parasites that show significant relatedness. Seventy-four per cent of parasite isolates showed ps > 0.3556 to one or more other parasites in the dataset, suggesting strong relatedness structure. Thirty-six per cent of these parasites were CI (ps = 0.95–1.00) to one or more other parasites in the dataset. Our simulations suggest that parasites that show significant relatedness but are not CI are either derived from the same zygote (0.5317 < ps < 0.7425) or share one common parent (0.3474 < ps < 0.5616).

### (b) Heritability of *in vitro* resistance

**Figure 2.**
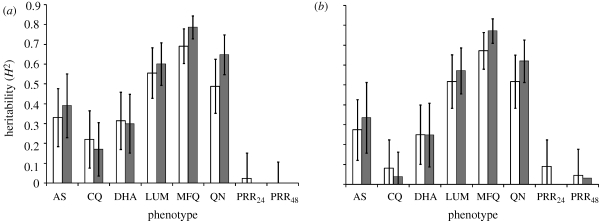
Heritability of *in vitro* drug resistance and parasite clearance. White bars show *H*^2^ estimated by ANOVA, while shaded bars show *H*^2^ estimates using variance components. Results are reported (*a*) using log-transformed IC_50_ data or clearance data (PRR_24_ and PRR_48_) and (*b*) for residuals following regression against patient age, gender and treatment category (see text). *H*^2^ estimates derived by both methods are very similar.

IC_50_ data for the six drugs is summarized in table 1. *H*^2^ estimates for IC_50_ values are shown in table 2 and figure 2. We found significant *H*^2^ for five of the six drugs using VC methods. Three drugs (MFQ, QN and LUM) showed high *H*^2^ (0.60–0.79, *p* = 4.8 × 10^−5^ to 7.6 × 10^−9^), while the three other drugs (CQ, DHA and AS) showed more marginal *H*^2^ (0.17–0.39, *p* = 0.02–0.08). Analysis of residuals to remove the effect of covariates (treatment regimen, patient age and sex) generated marginally lower *H*^2^ for all six drugs. Analysis of the same dataset using the ANOVA method (using log-transformed data or residuals) also gave similar results, with all drugs except CQ showing significant *H*^2^. The estimates of *H*^2^ were marginally lower for four of the six drugs using ANOVA compared with variance components, with an average difference of 0.07 (0.01–0.16).

**Table 1.** Summary of inhibitory concentration (IC_50_) for six antimalarial drugs.

	AS (nM)	CQ (nM)	DHA (nM)	LUM (nM)	MFQ (nM)	QN (nM)
min	0.40	48.0	0.30	4.0	6.10	79.20
max	11.60	918.5	28.50	157.9	353.10	1862.10
mean	2.25	256.29	3.55	48.56	82.21	746.68
median	1.9	210.8	2.7	40.3	70.1	650.8
*n*	152	175	165	155	176	152

**Table 2.** Heritability estimates using variance components and ANOVA-based methods. *p*-values are shown in italics (*p* < 0.001) and in bold (*p* < 0.05).

	clones	*n*	ln transformed		*p*	residuals^a^		*p*
			*H*^2^	s.e.		*H*^2^	s.e.	
variance components								
AS	24	151	0.39	0.16	**0.0174**	0.34	0.18	**0.0406**
CQ	25	174	0.17	0.13	0.08	0.04	0.12	0.3718
DHA	25	164	0.30	0.15	**0.0278**	0.25	0.16	0.0666
LUM	20	154	0.60	0.11	*4.84 × 10^−5^*	0.57	0.12	*0.0002*
MFQ	26	175	0.79	0.06	*2.85 × 10^−9^*	0.77	0.06	*1.47 × 10^−8^*
QN	20	151	0.65	0.10	*7.56 × 10^−5^*	0.62	0.11	*8.50 × 10^−5^*
PRR_24_	27	184	0	^b^	—	0.00	0.00	0.5
PRR_48_	27	184	0	^b^	—	0.03	0.10	0.3704
ANOVA								
AS	24	61^c^	0.33	0.15	**0.0123**	0.27	0.15	**0.0339**
CQ	25	66^c^	0.22	0.14	0.0551	0.08	0.14	0.2730
DHA	25	64^c^	0.31	0.14	**0.0143**	0.25	0.15	**0.0434**
LUM	20	54^c^	0.56	0.13	*<0.0001*	0.52	0.14	*0.0003*
MFQ	26	69^c^	0.69	0.09	*<0.0001*	0.67	0.09	*<0.0001*
QN	20	55^c^	0.49	0.14	*0.0004*	0.52	0.13	*0.0002*
PRR_24_	27	74^c^	0.02	0.13	0.4148	0.09	0.13	0.2377
PRR_48_	27	74^c^	−0.02	0.13	0.5408	0.05	0.13	0.3514

^a^Residuals were generated from multiple regression of phenotype against treatment regimen, patient age and gender.

^b^Boundary of parameter space encountered: s.e. and *p*-value not output by SOLAR.

^c^*n* is lower for the ANOVA as only CI parasites are used.

### (c) Heritability of CR

**Figure 3.**
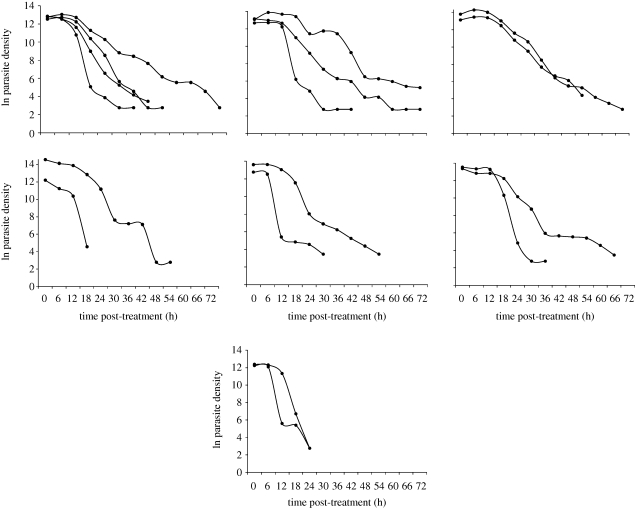
Clearance curves for groups of genetically similar parasite clones. The graphs show plots of parasite density sampled at 6-hourly intervals post-treatment. Parasite density is plotted on a natural log scale, which linearizes the decay curves. In many cases, CI parasites show dramatic differences in clearance profiles.

We calculated *H*^2^ of PRRs (PRR_24_ and PRR_48_). These calculations were performed on the natural logs of the phenotype data, as well as on the residuals following removal of the effects of gender, age and treatment type. *H*^2^ estimates derived from both simple ANOVA and more complex VC methods are shown in table 2 and figure 3. We found non-significant *H*^2^ for both PRR_24_ and PRR_48_ (*H*^2^ = 0 − 0.09, ns) using both estimation methods and using analysis with and without covariate effects.

There were 61 patients for whom 6-hourly measures of parasite density were made allowing estimation of first-order CR. Slopes for parasite density against time post-treatment ranged from 0.12 to 0.43 (mean (±1 s.d.) = 0.24 ± 0.07) and fitted well with a linear model (*r*^2^ = 0.90 ± 0.06; figure 3). There were seven CI parasite genotypes recovered from two to four different patients each. We found dramatic differences in clearance profile between many CI parasites (figure 3), consistent with the non-significant *H*^2^ for clearance-related parameters. Furthermore, statistical analysis using both VC- and ANOVA-based methods did not reveal significant effects of parasite genotype on CR (ANOVA: *H*^2^=0.16, n.s.; VC: *H*^2^=0.02 ± 0.25, *p* = 0.47), and remained insignificant after correction for age, sex and treatment type (ANOVA: *H*^2^=0.17, n.s.; VC: *H*^2^=0.03 ± 0.25, *p* = 0.46).

## 4. Discussion

These data demonstrate that (i) Thai parasite populations show strong relatedness structure, (ii) *H*^2^ can be effectively measured in natural parasite populations using inferred relatedness, (iii) *in vitro* drug resistance is strongly heritable and (iv) parasite CR following ACTs is not strongly influenced by parasite genotype on the Thailand–Burma border. We divide the discussion into three sections. First, we discuss the efficacy of two methods for measuring *H*^2^. Second, we evaluate the implications of these results for genetic mapping of ART resistance. Finally, we discuss the utility of *H*^2^ estimation in malaria.

### (a) Heritability estimation

We used 335 microsatellite markers distributed across the genome to estimate relationships between 185 parasites and used two methods to estimate *H*^2^. The genetic data reveal strong relatedness structure in the data. However, given the number and allelic diversity of the microsatellites genotyped, we were surprised that pairwise relatedness plots (figure 1*b*,*c*) did not clearly demarcate common relatedness categories. There are two likely reasons for this. First, gamete fusion between related parasites (i.e. inbreeding) may result in IBS information poorly defining relatedness categories. Second, the two parasite genomes that fuse to form a zygote may be unequally represented in the meiotic products. This has been well documented in *Plasmodium* genetic crosses in the laboratory, where selection results in over-representation of one of the two parental genotypes (Walker-Jonah *et al*. 1992). Hence, this parasite system is rather different from systems in which diploid individuals inherit one-half of their genome from each parent.

**Figure 4.**
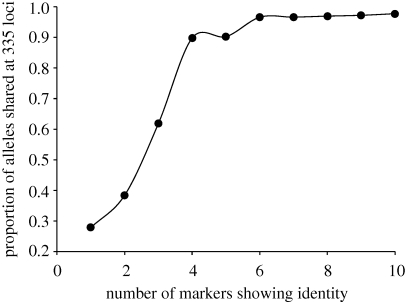
Number of loci required to identify CI parasites. We plotted the proportion of shared alleles (ps) between pairwise combinations of parasites using all 335 loci for parasites that share identity for a subsample of 1–10 sampled loci. The 10 sampled loci were randomly chosen from across the genome and had a mean expected heterozygosity of 0.80 (range = 0.52–0.91). Pairs of parasites that that were identical at more than six sampled loci showed greater than 95 per cent similarity across the genome. Hence, less than 10 loci effectively identify CI parasites.

We were extremely successful in measuring heritability using inferred relatedness. Encouragingly, both the VC method, using matrices of inferred relatedness between all parasites, and ANOVA methods that use only CI parasites gave very similar estimates of *H*^2^. The reason for this comes from inspection of patterns of relatedness. Thirty-six per cent of parasites sampled were CI with one or more other parasites. Because CI parasites are easily identified and the relationships between them are unambiguous, the ANOVA method effectively captures the relatedness class that is maximally informative for *H*^2^. A practical advantage of the ANOVA approach is that CI parasites can be identified with very few loci—in this data as few as six loci are sufficient (figure 4)—so large numbers of parasites can be rapidly screened. In contrast, the VC approach makes fuller use of the data because more distantly related parasites are included in *H*^2^ estimation. However, as distantly related parasites are on average less informative for *H*^2^, and relatedness estimates for such parasites show high variance (Csillery *et al*. 2006) and may be prone to bias owing to population structure (Shikano 2008), this does not greatly improve precision of *H*^2^ estimates. As noted in the introduction, the ANOVA method estimates the *broad-sense* heritability (*H*^2^) owing to additive and/or epistatic genetics, while the VC approach explicitly models narrow-sense heritability (*h*^2^). The close correspondence between measures derived from both VC and ANOVA approaches strongly suggests that additive variation is a principal determinant of phenotypic variation at the traits studied and that epistasis makes little contribution.

We used a simple allele-sharing measure to infer relatedness between parasites. This metric has the virtue of simplicity, but does not take into account background allele sharing that occurs by chance between unrelated parasites. We also computed kinship coefficients between pairs of parasites following Ritland (1996) using the program SPAGeDi (Hardy & Vekemans 2002), which accounts for background allele sharing. Reassuringly, we found that simple allele sharing (ps) and Ritland's more complex metric give extremely similar estimates of *H*^2^ (data not shown). The lack of bias may be due in part to the size of the dataset used.

Drug resistance is known to have a strong genetic basis (Hayton & Su 2004) and provides a positive control for our methods. Genetics explains a large proportion (49–79%) of the variance in IC_50_ values for LUM, QN and MFQ, but between 17 and 39 per cent of the variance for AS, DHA and CQ. By implication, factors other than genetics explain much variation in these drugs. The low *H*^2^ estimate of CQ response seems especially surprising given that the major gene determining resistance, the chloroquine resistance transporter (*pfcrt*), is well characterized (Fidock *et al*. 2000). However, the *pfcrt-76T* SNP conferring resistance is fixed on the Thailand–Burma border, so all parasites show high IC_50_. The low *H*^2^ estimate suggests that much of the remaining variation in IC_50_ measures observed does not have a genetic basis. We list possible non-genetic explanations for the variation in IC_50_ observed:

—The assays of IC_50_ were conducted in blood samples collected from patients. Differences in red blood cell physiology between patients, such as permeability to drugs, may influence assay results.
—Experimental error in preparation of drug plates may add noise to the data and contribute to the unexplained variation. Laboratory-based studies of drug resistance generally repeat drug assays multiple times to maximize accuracy (Ferdig *et al*. 2004). Unfortunately this is not possible in the field situation when isolates are processed fresh from the patient without culture adaptation.
—Epigenetic modifications could affect phenotype measures resulting in non-genetic differences between parasites (Slatkin 2009). Currently, little is known about epigenetic effects in *P. falciparum* for traits other than *var* gene expression (Volz *et al*. 2010): the role of epigenetics is unknown for the phenotypes examined here.

### (b) CR is not significantly heritable on the Thai–Burma border

We examined clearance following ART as an example of a trait that cannot be measured in the laboratory, but is of considerable biomedical interest. Slow clearance of parasites following treatment with ACTs has been widely interpreted as a sign of impending resistance to artemisinin derivatives (White 2008; Carrara *et al*. 2009; Dondorp *et al*. 2009). While we observed extensive variation in clearance parameters (PRR_24_, PRR_48_ and CR), these data provided no evidence that these measures are influenced by parasite genetic factors on the Thailand–Burma border. There are two possible explanations for this result. First, our measures of clearance may not be sufficiently accurate to detect significant *H*^2^, because we recorded parasite density at 24 h intervals for most patients. More frequent measures may be needed to accurately document CR. We note that detailed (6-hourly) CR information was available for a subset of 61 patients. However, this subsample also failed to detect significant *H*^2^ for clearance.

Second, clearance may truly have little genetic basis and is determined by factors other than parasite genetics in this population. What might these other factors be? We evaluated three possible factors—patient age (a surrogate measure of immunity), patient gender and treatment regimen. However, of these three, only gender marginally influenced PRR_24_. Mathematical modelling work suggests that the age structure of parasite populations within the patient at the time of treatment may significantly influence clearance profiles (L. White 2007, personal communication), as different life stages vary in their response to artemisinin derivatives. Similarly, heterogeneity in patient immune status as a consequence of exposure to infection could influence clearance patterns (Luxemburger *et al*. 1997), while human genetic factors could also play a role (Weatherall 2008). The key point here is that many factors other than parasite genotype may influence CR.

It is important to note that these results apply specifically to parasite populations sampled from the Thailand–Burma border between 2000 and 2003. Carrara *et al*. (2009) show that there has been a decrease in CR in this region, but that this did not start until 2003–2004. As the parasites examined here were sampled prior to 2003, it is possible that genes influencing clearance have spread to the Thailand–Burma border since this time. We have recently examined *H*^2^ for CR in a population in western Cambodia, where extremely slow clearance and high failure rates have been reported (Dondorp *et al*. 2009). Interestingly, we observed high *H*^2^ in western Cambodia (0.56–0.58), clearly implicating parasite genetic factors (Anderson *et al*. 2010). We suggest larger studies are required on the Thailand–Burma border, entailing 6-hourly measures of clearance, to determine whether parasite clearance has a genetic basis in present-day parasite populations.

### (c) Applications of *H*^2^ for malaria research

Our ability to rapidly genotype or sequence malaria genomes now make GWASs an attractive alternative to linkage mapping for locating genes that underlie traits of biomedical or biological importance (Su *et al*. 2007). As such studies are expensive, it is important to first demonstrate that the traits of interest have a significant genetic basis. *H*^2^ estimates provide one way to achieve this. We provide one example of a trait of enormous public health significance that does not have a genetic basis in the population studied: clearance following ART. GWASs of this trait would therefore be unlikely to succeed in this population. Virulence and sex ratio are two other traits where the role of parasite genetics is uncertain, yet both are envisaged as adaptive traits with a genetic basis (West *et al*. 2001; Mackinnon & Read 2004). Studies of *H*^2^ would provide a test for such models and would provide preliminary data to justify GWASs on such traits. To extend this argument further, the magnitude of *H*^2^ is also useful for estimating sample sizes for GWASs, as traits with strong *H*^2^ require smaller sample sizes than traits with weak *H*^2^. Typically, sample size scales with the square of *H*^2^, so *n* for a trait with *H*^2^ = 1 is four times less than *n* for a trait with *H*^2^ = 0.5 (Williams & Blangero 1999). However, we note that trait architecture is also critically important in determining mappability. True polygenic traits determined by many genes of small effect size may be difficult to map even if they show high *H*^2^ (Goring *et al*. 2007).

*H*^2^ measures are also useful for assessing the robustness of phenotype measures that are known to have a strong genetic basis. In laboratory studies, precision of phenotypes can be directly assessed by repeated measurement. For many malaria traits, such repeated measurement is not feasible. However, as related parasites are sampled within populations, estimating *H*^2^ provides an alternative approach to assessing robustness of phenotype measures. The IC_50_ data presented here, which were measured directly using parasite-infected blood from patients, provides an example of this application. Our results indicate that IC_50_ data collected in this way showed poor repeatability for three or six drugs examined.

Our ability to measure *H*^2^ is strongly dependent on the existence of related or identical parasites within population samples. In *P. falciparum*, the proportion of CI parasites within populations is dependent on levels of transmission and inbreeding (Anderson *et al*. 2000). In low-transmission regions, multiple clone infections are rare, simplifying genotype–phenotype association. Hence, measurement of *H*^2^ is easiest in low-transmission regions such as southeast Asia and South America. However, even in high-transmission African countries, a large proportion of meioses are expected to result from inbreeding (Babiker *et al*. 1994; Razakandrainibe *et al*. 2005). CI genotypes may also be sampled in such regions (Conway & McBride 1991), and could be used to estimate *H*^2^.

## References

[RSPB20100196C1] Anderson T. J. 2000 Microsatellite markers reveal a spectrum of population structures in the malaria parasite *Plasmodium falciparum*. Mol. Biol. Evol. 17, 1467–1482 11018154 10.1093/oxfordjournals.molbev.a026247

[RSPB20100196C2] Anderson T. J. Nair S. Qiu W. G. Singlam S. Brockman A. Paiphun L. Nosten F. 2005 Are transporter genes other than the chloroquine resistance locus (*pfcrt*) and multi drug resistance gene (*pfmdr*) associated with antimalarial drug resistance? Antimicrob. Agents Chemother. 272, 1153–1161 10.1128/AAC.49.6.2180-2188.2005 PMC1140548 15917511

[RSPB20100196C3] Anderson T. J. 2010 High heritability of malaria parasite clearance rate indicates a genetic basis for artemisinin resistance in Western Cambodia. J. Infect. Dis. 201, 1326–1330 (doi:10.1086/651562) 20350192 10.1086/651562 PMC2853733

[RSPB20100196C4] Babiker H. A. Ranford-Cartwright L. C. Currie D. Charlwood J. D. Billingsley P. Teuscher T. Walliker D. 1994 Random mating in a natural population of the malaria parasite *Plasmodium falciparum*. Parasitology 109, 413–421 7800409 10.1017/s0031182000080665

[RSPB20100196C5] Blangero J. Williams J. T. Almasy L. 2001 Variance component methods for detecting complex trait loci. Adv. Genet. 42, 151–181 (doi:10.1016/S0065-2660(01)42021-9) 11037320 10.1016/s0065-2660(01)42021-9

[RSPB20100196C6] Blouin M. S. 2003 DNA-based methods for pedigree reconstruction and kinship analysis in natural populations. Trends Ecol. Evol. 18, 503–511 (doi:10.1016/S0169-5347(03)00225-8)

[RSPB20100196C7] Blouin M. S. Parsons M. Lacaille V. Lotz S. 1996 Use of microsatellite loci to classify individuals by relatedness. Mol. Ecol. 5, 393–401 8688959 10.1111/j.1365-294x.1996.tb00329.x

[RSPB20100196C8] Brockman A. 2000 *Plasmodium falciparum* antimalarial drug susceptibility on the north-western border of Thailand during five years of extensive use of artesunate–mefloquine. Trans. R. Soc. Trop. Med. Hyg. 94, 537–544 (doi:10.1016/S0035-9203(00)90080-4) 11132385 10.1016/s0035-9203(00)90080-4 PMC4340572

[RSPB20100196C9] Carrara V. I. 2009 Changes in the treatment responses to artesunate–mefloquine on the northwestern border of Thailand during 13 years of continuous deployment. PLoS ONE 4, e4551 (doi:10.1371/journal.pone.0004551) 19234601 10.1371/journal.pone.0004551 PMC2641001

[RSPB20100196C10] Conway D. J. McBride J. S. 1991 Genetic evidence for the importance of interrupted feeding by mosquitoes in the transmission of malaria. Trans. R. Soc. Trop. Med. Hyg. 85, 454–456 (doi:10.1016/0035-9203(91)90217-M) 1755049 10.1016/0035-9203(91)90217-m

[RSPB20100196C11] Csillery K. 2006 Performance of marker-based relatedness estimators in natural populations of outbred vertebrates. Genetics 173, 2091–2101 (doi:10.1534/genetics.106.057331) 16783017 10.1534/genetics.106.057331 PMC1569738

[RSPB20100196C12] Desjardins R. E. Canfield C. J. Haynes J. D. Chulay J. D. 1979 Quantitative assessment of antimalarial activity *in vitro* by a semiautomated microdilution technique. Antimicrob. Agents Chemother. 16, 710–718 394674 10.1128/aac.16.6.710 PMC352941

[RSPB20100196C13] Dondorp A. M. 2009 Artemisinin resistance in *Plasmodium falciparum* malaria. N. Engl. J. Med. 361, 455–467 (doi:10.1056/NEJMoa0808859) 19641202 10.1056/NEJMoa0808859 PMC3495232

[RSPB20100196C47] Doumbo O. K. Thera M. A. Koné A. K. Raza A. Tempest L. J. Lyke K. E. Plowe C. V. Rowe J. A. 2009 High levels of *Plasmodium falciparum* rosetting in all clinical forms of severe malaria in African children. Am. J. Trop. Med. Hyg. 81, 987–993 (doi:10.4269/ajtmh.2009.09-0406) 19996426 10.4269/ajtmh.2009.09-0406 PMC2877664

[RSPB20100196C14] Falconer D. S. Mackay T. F. 1996 Introduction to quantitative genetics Harlow, UK: Prentice-Hall 10.1093/genetics/167.4.1529 PMC1471025 15342495

[RSPB20100196C48] Felsenstein J. 1993 PHYLIP *(*phylogeny inference package*)*, v. 3.57 Seattle, WA: University of Washington

[RSPB20100196C15] Ferdig M. T. Cooper R. A. Mu J. Deng B. Joy D. A. Su X. Z. Wellems T. E. 2004 Dissecting the loci of low-level quinine resistance in malaria parasites. Mol. Microbiol. 52, 985–997 (doi:10.1111/j.1365-2958.2004.04035.x) 15130119 10.1111/j.1365-2958.2004.04035.x

[RSPB20100196C16] Fernandez J. Toro M. A. 2006 A new method to estimate relatedness from molecular markers. Mol. Ecol. 15, 1657–1667 (doi:10.1111/j.1365-294X.2006.02873.x) 16629818 10.1111/j.1365-294X.2006.02873.x

[RSPB20100196C17] Fidock D. A. 2000 Mutations in the *P. falciparum* digestive vacuole transmembrane protein PfCRT and evidence for their role in chloroquine resistance. Mol. Cell 6, 861–871 (doi:10.1016/S1097-2765(05)00077-8) 11090624 10.1016/s1097-2765(05)00077-8 PMC2944663

[RSPB20100196C18] Fidock D. A. Eastman R. T. Ward S. A. Meshnick S. R. 2008 Recent highlights in antimalarial drug resistance and chemotherapy research. Trends Parasitol. 24, 537–544 (doi:10.1016/j.pt.2008.09.005) 18938106 10.1016/j.pt.2008.09.005 PMC2718548

[RSPB20100196C19] Goring H. H. 2007 Discovery of expression QTLs using large-scale transcriptional profiling in human lymphocytes. Nat. Genet. 39, 1208–1216 (doi:10.1038/ng2119) 17873875 10.1038/ng2119

[RSPB20100196C21] Hardy O. J. Vekemans X. 2002 SPAGeDi: a versatile computer program to analyse spatial genetic structure at the individual or population levels. Mol. Ecol. Notes 2, 618–620 (doi:10.1046/j.1471-8286.2002.00305.x)

[RSPB20100196C22] Havill L. M. 2010 Heritability of lumbar trabecular bone mechanical properties in baboons. Bone 46, 835–840 (10.1016/j.bone.2009.11.002). 19900599 10.1016/j.bone.2009.11.002 PMC3005696

[RSPB20100196C23] Hayton K. Su X. Z. 2004 Genetic and biochemical aspects of drug resistance in malaria parasites. Curr. Drug Targets. Infect. Disord. 4, 1–10 (doi:10.2174/1568005043480925) 15032630 10.2174/1568005043480925

[RSPB20100196C24] Klaper R. Ritland K. Mousseau T. A. Hunter M. D. 2001 Heritability of phenolics in *Quercus laevis* inferred using molecular markers. J. Hered. 92, 421–426 (doi:10.1093/jhered/92.5.421) 11773250 10.1093/jhered/92.5.421

[RSPB20100196C25] Kruuk L. E. Clutton-Brock T. H. Slate J. Pemberton J. M. Brotherstone S. Guinness F. E. 2000 Heritability of fitness in a wild mammal population. Proc. Natl Acad. Sci. USA 97, 698–703 (doi:10.1073/pnas.97.2.698) 10639142 10.1073/pnas.97.2.698 PMC15393

[RSPB20100196C26] Luxemburger C. Ricci F. Nosten F. Raimond D. Bathet S. White N. J. 1997 The epidemiology of severe malaria in an area of low transmission in Thailand. Trans. R. Soc. Trop. Med. Hyg. 91, 256–262 (doi:10.1016/S0035-9203(97)90066-3) 9231189 10.1016/s0035-9203(97)90066-3

[RSPB20100196C27] Lynch M. Walsh B. 1998 Genetics and analysis of quantitative traits, pp. 581–595 Sunderland, MA: Sinauer Associates

[RSPB20100196C28] Mackinnon M. J. Read A. F. 2004 Virulence in malaria: an evolutionary viewpoint. Phil. Trans. R. Soc. Lond. B 359, 965–986 (doi:10.1098/rstb.2003.1414) 15306410 10.1098/rstb.2003.1414 PMC1693375

[RSPB20100196C29] Mackinnon M. J. Gaffney D. J. Read A. F. 2002 Virulence in rodent malaria: host genotype by parasite genotype interactions. Infect. Genet. Evol. 1, 287–296 (doi:10.1016/S1567-1348(02)00039-4) 12798007 10.1016/s1567-1348(02)00039-4

[RSPB20100196C30] Paul R. E. Packer M. J. Walmsley M. Lagog M. Ranford-Cartwright L. C. Paru R. Day K. P. 1995 Mating patterns in malaria parasite populations of Papua New Guinea. Science 269, 1709–1711 (doi:10.1126/science.7569897) 7569897 10.1126/science.7569897

[RSPB20100196C31] Razakandrainibe F. G. Durand P. Koella J. C. de Meeus T. Rousset F. Ayala F. J. Renaud F. 2005 ‘Clonal’ population structure of the malaria agent *Plasmodium falciparum* in high-infection regions. Proc. Natl Acad. Sci. USA 102, 17 388–17 393 (doi:10.1073/pnas.0508871102) 10.1073/pnas.0508871102 PMC1297693 16301534

[RSPB20100196C32] Read A. F. Narara A. Nee S. Keymer A. E. Day K. P. 1992 Gametocyte sex ratios as indirect measures of outcrossing rates in malaria. Parasitology 104, 387–395 1641238 10.1017/s0031182000063630

[RSPB20100196C33] Reilly H. B. Wang H. Steuter J. A. Marx A. M. Ferdig M. T. 2007 Quantitative dissection of clone-specific growth rates in cultured malaria parasites. Int. J. Parasitol. 37, 1599–1607 (doi:10.1016/j.ijpara.2007.05.003) 17585919 10.1016/j.ijpara.2007.05.003 PMC2268714

[RSPB20100196C34] Ritland K. 1996 Marker-based method for inferences about quantitative inheritance in natural populations. Evolution 50, 1062–1073 (doi:10.2307/2410647) 10.1111/j.1558-5646.1996.tb02347.x 28565279

[RSPB20100196C35] Ritland K. 2000 Marker-inferred relatedness as a tool for detecting heritability in nature. Mol. Ecol. 9, 1195–1204 (doi:10.1046/j.1365-294x.2000.00971.x) 10972759 10.1046/j.1365-294x.2000.00971.x

[RSPB20100196C36] Shikano T. 2008 Estimation of quantitative genetic parameters using marker-inferred relatedness in Japanese flounder: a case study of upward bias. J. Hered. 99, 94–104 (doi:10.1093/jhered/esm105) 18209112 10.1093/jhered/esm105

[RSPB20100196C37] Slatkin M. 2009 Epigenetic inheritance and the missing heritability problem. Genetics 182, 845–850 (doi:10.1534/genetics.109.102798) 19416939 10.1534/genetics.109.102798 PMC2710163

[RSPB20100196C38] Su X. Hayton K. Wellems T. E. 2007 Genetic linkage and association analyses for trait mapping in *Plasmodium falciparum*. Nat. Rev. Genet. 8, 497–506 (doi:10.1038/nrg2126) 17572690 10.1038/nrg2126

[RSPB20100196C39] Thomas S. C. Pemberton J. M. Hill W. G. 2000 Estimating variance components in natural populations using inferred relationships. Heredity 84, 427–436 10849066 10.1046/j.1365-2540.2000.00681.x

[RSPB20100196C40] Thompson E. A. 1974 Gene identities and multiple relationships. Biometrics 30, 667–680 (doi:10.2307/2529231) 4429760

[RSPB20100196C41] Volz J. 2010 Potential epigenetic regulatory proteins localise to distinct nuclear sub-compartments in *Plasmodium falciparum*. Int. J. Parasitol 40, 109–121 19765590 10.1016/j.ijpara.2009.09.002

[RSPB20100196C42] Walker-Jonah A. Dolan S. A. Gwadz R. W. Panton L. J. Wellems T. E. 1992 An RFLP map of the *Plasmodium falciparum* genome, recombination rates and favored linkage groups in a genetic cross. Mol. Biochem. Parasitol. 51, 313–320 1349423 10.1016/0166-6851(92)90081-t

[RSPB20100196C43] Weatherall D. J. 2008 Genetic variation and susceptibility to infection: the red cell and malaria. Br. J. Haematol. 141, 276–286 18410566 10.1111/j.1365-2141.2008.07085.x

[RSPB20100196C44] West S. A. Reece S. E. Read A. F. 2001 Evolution of gametocyte sex ratios in malaria and related apicomplexan (protozoan) parasites. Trends Parasitol. 17, 525–531 (doi:10.1016/S1471-4922(01)02058-X) 11872397 10.1016/s1471-4922(01)02058-x

[RSPB20100196C45] White N. J. 2008 Qinghaosu (artemisinin): the price of success. Science 320, 330–334 (doi:10.1126/science.1155165) 18420924 10.1126/science.1155165

[RSPB20100196C46] Williams J. T. Blangero J. 1999 Power of variance component linkage analysis to detect quantitative trait loci. Ann. Hum. Genet. 63, 545–563 (doi:10.1046/j.1469-1809.1999.6360545.x) 11246457 10.1017/S0003480099007848

